# Habitat Use and Activity Patterns of Mammals and Birds in Relation to Temperature and Vegetation Cover in the Alpine Ecosystem of Southwestern China with Camera-Trapping Monitoring

**DOI:** 10.3390/ani11123377

**Published:** 2021-11-25

**Authors:** Zhouyuan Li, Zhuo Tang, Yanjie Xu, Yingying Wang, Zhaogang Duan, Xuehua Liu, Pengyan Wang, Jian Yang, Wei Chen, Herbert H. T. Prins

**Affiliations:** 1 State Key Joint Laboratory of Environment Simulation and Pollution Control, School of Environment, Tsinghua University, Beijing 100084, China; lizhouyuan@bjfu.edu.cn (Z.L.); 84039005@163.com (Z.T.); 2 China Grassland Research Center, School of Grassland Science, Beijing Forestry University, Beijing 100083, China; 3 Administration Bureau of Wolong National Nature Reserve, Wenchuan 623006, China; ddgryx@163.com; 4 The Finnish Museum of Natural History, University of Helsinki, P.O. Box 17, 00100 Helsinki, Finland; yanjie.xu@helsinki.fi; 5 Wildlife Ecology and Conservation Group, Wageningen University & Research, 6708 PB Wageningen, The Netherlands; yingying.xg.wang@jyu.fi; 6 China Conservation and Research Center for the Giant Panda, Dujiangyan 611830, China; wolongmuseum@163.com; 7 School of Resources and Environmental Engineering, Anhui University, Hefei 230601, China; wchen1949@163.com; 8 Animal Sciences Group, Wageningen University & Research, 6708 WD Wageningen, The Netherlands; Herbert.Prins@wur.nl

**Keywords:** biodiversity, environmental factor, camera trapping, mountain, wildlife

## Abstract

**Simple Summary:**

The Wolong National Nature Reserve in Sichuan province covers a unique mountainous ecosystem located on the eastern border of the Tibetan Plateau in China. We applied a popular non-invasive observational method, i.e., infrared-triggered camera trapping, to gain thousands of photographs of wildlife to monitor biodiversity over three years. Combined with data on the local abiotic factors, our integrative statistical analysis identified the key environmental drivers, i.e., temperature and vegetation, affecting the distribution and abundance of mammals and birds in the reserve. All species were classified into three main types by their tolerance of or fondness for different environmental conditions. The detectability of each species by camera trapping was quantified and ranked to provide insights on each species’ relative abundance in the area.

**Abstract:**

The high-altitude ecosystem of the Tibetan Plateau in China is a biodiversity hotspot that provides unique habitats for endemic and relict species along an altitudinal gradient at the eastern edge. Acquiring biodiversity information in this area, where the average altitude is over 4000 m, has been difficult but has been aided by recent developments in non-invasive technology, including infrared-triggered camera trapping. We used camera trapping to acquire a substantial number of photographic wildlife records in Wolong National Nature Reserve, Sichuan, China, from 2013 to 2016. We collected information of the habitat surrounding the observation sites, resulting in a dataset covering 37 species and 12 environmental factors. We performed a multivariate statistical analysis to discern the dominant environmental factors and cluster the mammals and birds of the ecosystem in order to examine environmental factors contributing to the species’ relative abundance. Species were generalized into three main types, i.e., cold-resistant, phyllophilic, and thermophilic, according to the identified key environmental drivers (i.e., temperature and vegetation) for their abundances. The mammal species with the highest relative abundance were bharal (*Pseudois nayaur*), Moupin pika (*Ochotona thibetana*), and Himalayan marmot (*Marmota himalayana*). The bird species with highest relative abundance were snow partridge (*Lerwa lerwa*), plain mountain finch (*Leucosticte nemoricola*), Chinese monal (*Lophophorus lhuysii*), and alpine accentor (*Prunella collaris*).

## 1. Introduction

The main problems that constrain the efficacy of biodiversity conservation in China include habitat loss and fragmentation, overuse and environmental pollution. The underlying causes are unsustainable economic development and the pressures of land development that reduce biodiversity [1]. It is important to monitor wildlife and categorize the collected information on the multi-environmental gradient axes to perform better fitted conservation strategies. Due to the unique geographical and climatic conditions on the border of the Qinghai–Tibet Plateau, researchers have realized the importance of its biodiversity. The region provides quaternary glacial refugia for species, which has been investigated in many studies of ecological observation and biological conservation in the Wolong National Nature Reserve (WNNR; Sichuan Province, PR China). The high-altitude ecosystem of the eastern Qinghai–Tibet Plateau has a profound elevational gradient with high vertical uplift distance in a relatively limited space. Mammals and birds face severe challenges relative to habitat loss due to climate change and human interference, especially for mammals living at high altitudes, which have the highest proportion of threatened species [2,3]. Mammal diversity is an important part of biodiversity and is often used as an indicator of habitat quality and changes to provide an objective and measurable basis for biodiversity research [4].

Under the pressure of population growth and urbanization, habitat loss has become more severe in recent decades as the landscape fragments [5]. The Chinese government made efforts to balance the land development and nature conservation by establishing nature reserves and creating an ecological ‘red-line’, i.e., a legally restricted area of land that is strictly prohibited to be farmed or developed [6] Biodiversity conservation requires the creation of national parks to protect wildlife habitats, which is a recent plan launched by the national government [7]. The designation and prioritization of such plans require the monitoring of mammals and their trends in populations, as well as those of other wildlife.

Apart from habitat loss, another major challenge for the wildlife in China is climate change, especially in high-altitude mountainous areas where the limited vertical space reduces the flexibility of migration or emigration as a consequence [2,8]. More knowledge of the various responses of wildlife to climatic factors is needed, especially in the least developed and most depopulated areas, such as the mountainous areas of southwestern China. Under global warming, concerns over the biodiversity in alpine ecosystems are deeper than ever because of rapid climate change and acute consequences for local wildlife activities. Monitoring and generalizing the pattern of wildlife in alpine ecosystems is the first step to developing strategies to understand and manage threats with the aim to ensure the long-term persistence of species.

Previous investigations have mainly been carried out in the forest ecosystem of relatively low-altitude areas of the WNNR. Research on alpine mammals and birds has been minimal. Infrared-triggered camera trapping, a non-invasive and highly efficient observational technique, has been popular compared to conventional tools such as wireless telemetry collars [9,10]. This technology has facilitated continuous observations of alpine wildlife activities in the southwestern mountains of the Qinghai–Tibet Plateau [11,12]. In this paper, we applied infrared-triggered camera trapping to monitor the biodiversity in the WNNR, mainly aiming to identify (1) the species that occur in the reserve and their activity patterns and (2) the relationship between environmental factors and species occurrence/abundance. The results have implications for habitat improvement measures and provide a baseline for future wildlife research.

## 2. Materials and Methods

### 2.1. Study Area and Sampling Sites

**Figure 1:**
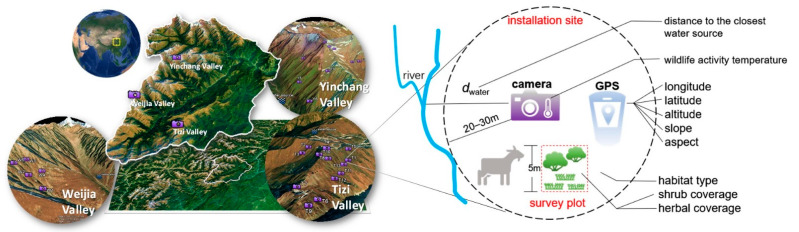
Topographic map with locations of the infrared-triggered camera installations, and a schematic diagram of the environmental factor acquisition in the study area, Wolong National Nature Reserve in Sichuan, China.

Between 2013 and 2016, a total of 27 Ltl-6210 MC infrared-triggered cameras was installed in the installation sites in the study area. Infrared-triggered camera trapping is a non-invasive method for monitoring the relative abundance of wildlife, with few interferences and relatively high efficiency. In practice, we first selected three valleys where wildlife was most frequently observed in the routine survey (Figure 1).

The elevation of the study area ranged from ~3500 to ~4500 m. Then, we designated installation sites within the chosen valleys based on the following criteria: (1) the different habitats were diversified so we aimed to cover all the different main alpine ecosystem types in the nature reserve, i.e., shrub, meadow, and scree; (2) we picked locations where the local topographical and geological conditions allowed us to set up a hidden and stable camera observation and recyclable device. The specific site selection to distribute the cameras mainly depended on the reachability, safety, and micro-environmental conditions in the field.

As a result, 27 sites, including 13 in Tizi Valley, 9 in Yinchang Valley, and 5 in Weijia Valley, were used for camera trapping (Figure 1). The elevation of these installation sites ranged from 3536 m to 4481 m. Cameras were set 40–50 cm above the ground in the tracks of wildlife (i.e., near traces of mammal scats and mammal or bird footprints), paths of beasts (i.e., the narrow passageways shaped by frequently passing beasts), and mountain ridges. The linear distances between cameras in the same valley ranged from ~100 m to ~500 m. An animal movement triggers a camera to take three photos over 20 s followed by a 10–20 s video. Every three months, we checked and collected the data captured by the cameras. During the monitoring period, four cameras were lost and three cameras were broken, which were replaced within three months.

### 2.2. Environmental Factors Measurement

We measured 12 environmental factors, encompassing geographical, vegetation, energy, and water environmental information, during camera installation in the trapping locations (Supplementary Table S2). We measured geographical and topographic metrics, including latitude, longitude, altitude, slope, and aspect, by a handheld GPS. The vegetational information, including species information, herbal coverage (0~100%), and shrub coverage (0~100%), were measured in an area of 5 m × 5 m within a radius of 20–30 m around the camera installation point (Figure 1). In the quantification of the habitat types, we integrated the herb coverage, shrub coverage, and community-dominant species information with the aim of transferring the categorical factor of the habitat types to numerical scores to reflect that the habitat selected was a continuous spectrum, naturally mixed with land cover of herbal grassland, shrub grassland, and nearly bare rocks—though most places at our study area were scree landscape. For evenness of distribution in the quantification, a formula was constructed to assess habitat types as follows:

$$\mathrm{score}\;=\;\{\begin{array}{l} 10\times \sqrt{\text{shrub coverage}\times W_{shub}+\text{herbal coverage}},\mathrm{without}\;\mathrm{the}\;\mathrm{dominant}\;\mathrm{species}\;\mathrm{of}\;\mathrm{alpine}\;\mathrm{meadow}\;\\ 10\times \sqrt{\text{shrub coverage}\times W_{shub}+\text{herbal coverage}}\;+\;1,\mathrm{without}\;\mathrm{the}\;\mathrm{dominant}\;\mathrm{species}\;\mathrm{of}\;\mathrm{alpine}\;\mathrm{meadow} \end{array}$$

where $W_{shub}$ is the weight of the shrub species in the community. The dominant species of alpine meadow refers to the plants belonging to the genuses *Pedicularis*, *Ligularia*, and *Care*, as these are the major components (over approximately 50%) of the community around the site. Based on the score, the four main habitat types were generalized and labeled ‘alpine shrub’ (score > 12), ‘alpine meadow’ (12 ≥ score > 9), ‘alpine scree–meadow’ transition zone (9 ≥ score > 6), and ‘alpine scree’ (score ≤ 6). The cameras recorded the ambient temperature as wildlife activity temperature when triggered to take photographs of wildlife. For the vegetational environment, the score of habitat type reflected comprehensive living conditions as an integrated indicator, while shrub or herbal coverage reflected how plants grew in and covered the survey site from a single aspect. Based on these records, maximum activity temperature, minimum activity temperature, and average activity temperature were calculated for the subsequent analysis. The linear distance from the camera installation sites to the closest water source was measured in the projected map on the QGIS platform with the geographical coordinates of the camera installation sites.

### 2.3. Calculation of Relative Abundance

The photos captured by the cameras were analyzed and the captured videos were used to assist species recognition. Photos of the same species from one camera within 30 min or consecutive photos of different species were defined as individual effective detections [13]. The relative abundance (*RA*) is positively proportional to the wildlife population sizes [11,12] and was calculated with the following formula:

*RA* = (*A*/*N*) × 1000 (1)

where *A* represents the number of efficient captures of certain species, and *N* represents the number of camera-working days. One camera-working day was defined when a camera worked continuously for 24 h. The *RA* was calculated for each species.

### 2.4. Environmental Factors and Species Analysis

Species’ *RA* and environmental factors were used for detrended correspondence canonical analysis (DCCA) to detect principal environmental factors and to cluster species [14]. The X- and Y-axis were added to indicate key clustering factors and to classify species.

## 3. Results

### 3.1. The Overall Biodiversity

**Figure 2:**
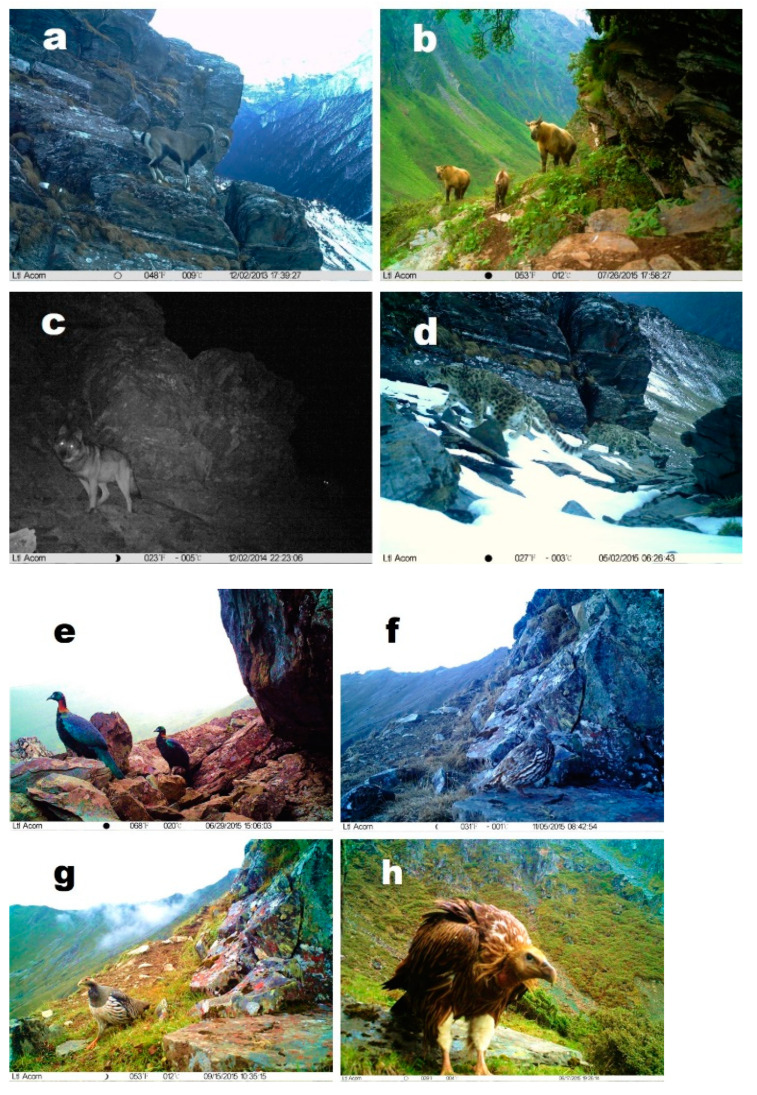
Representative photos of mammals and birds imaged by the infrared cameras in the Wolong National Nature Reserve. Bharal (**a**), Sichuan takin (**b**), wolf (*Canis lupus*) (**c**), snow leopard (**d**), Chinese monal (**e**), snow partridge (*Lerwa lerwa*) (**f**), Tibetan snowcock (**g**), and Himalayan vulture (**h**).

The cameras recorded for 7056 days and we retrieved ~90,000 photos and ~30,000 video clips, including 2251 effective detections (see the definition in Section 2) of wildlife, leading to the identification of 37 species (Figure 2). The invalid photographing (i.e., photos without wildlife detection) represented ~20% of all trigger actions. A total of four of the 37 photographed species are listed as category I species and seven as category II species in the Chinese National List of Protected Animals (Supplementary Table S1). Among them, the Sichuan takin (*Budorcas tibetanus*) and Chinese monal (*Lophophorus lhuysii*) are species endemic to China. Of the 37 photographed species, the snow leopard (*Panthera uncia*), Chinese monal, and beech marten (*Martes foina*) are endangered species in China according to the listing criteria of the Chinese National List of Protected Animals. The Sichuan takin and Chinese goral (*Naemorhedus griseus*) are vulnerable species, and we also identified 11 near-threatened species [15]. Other species, mostly small mammals and passerines, are listed as ‘Least Concern’ by the International Union for the Conservation of Nature (IUCN).

### 3.2. Environmental Factors Analysis and Species Clusters

**Figure 3:**
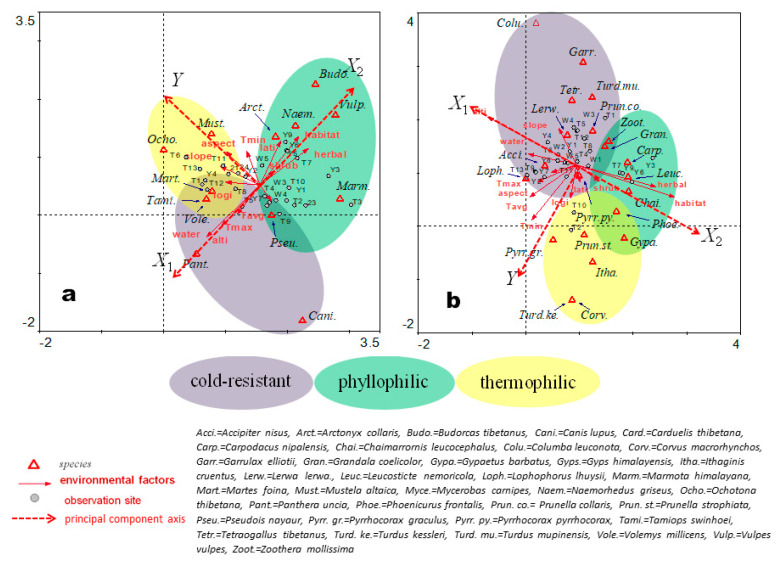
The results of the detrended canonical correspondence analysis (DCCA) with the clustered habitat preferences for mammals (**a**) and birds (**b**) by the gradient of environmental factors in a high-altitude ecosystem.

The results of the DCCA are shown in Figure 3. The 12 environmental factors orthogonal to each other form two principal component axes (X, Y), in which the environmental factor arrows are distributed in three main directions (X_1_, X_2_, and Y). The X-axis indicates the material environment information that changes with the elevation gradient, with X_1_ as a geographical factor and X_2_ as a vegetation factor. The Y-axis indicates the energy environment information that includes temperature-related factors and terrain gradients. The most dominant component of X_1_ is elevation, and other factors closely related to X_1_ include the distance between observation sites, water sources, and maximum activity temperature (*T*_max_ on the graphs). X_2_ includes herbal vegetation coverage, shrub coverage, and habitat type. The opposite directions of X_1_ and X_2_ indicate that alpine vegetation coverage decreases with an increase in elevation. The angles among the factors on the Y-axis are less than 90 degrees, indicating positive relationships between these factors. Aspect, slope, and minimum activity temperature (*T*_min_ on the graphs), which are close to each other, are relatively accordant with the principal component Y-axis (Figure 3a). In Figure 3b, aspect and minimum activity temperature are close and mostly accordant with the Y-axis, while slope could be partly projected to the reverse extension line of the Y-axis. Aspect and slope are related to energy balance by sunlight. Ambient temperature is directly related to the energy of the biological activity and metabolic processes of the individuals [16]. Therefore, the minimum activity temperature is one of the key environmental factors for the observed wildlife in alpine ecosystems.

According to the results of the DCCA analysis, species are categorized into three groups. The first group is defined as cold-resistant species (purple oval in Figure 3), which are distributed on the positive side of the X_1_-axis and around the opposite extension line of the Y-axis. This indicates that they are distributed in high-altitude areas and have a lower minimum activity temperature. The second group is defined as phyllophilic species, which react positively to foliage (green oval). These species are distributed on the positive side of the X_2_-axis, indicating that they are highly dependent on habitats with high vegetation cover at low altitudes. The third group is defined as thermophilic species, which tend to select warmer places (yellow oval). These species are distributed in the positive side of the Y-axis and have a relatively shorter projection on the X-axis, indicating that they have a higher minimum activity temperature and less sensitivity to environmental materials (i.e., vegetation and water resources).

### 3.3. Detection Probability of the Species Groups

**Figure 4:**
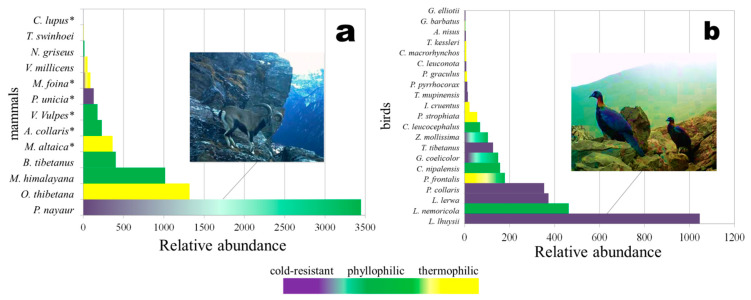
The relative abundance rankings of mammals (**a**) and birds (**b**) in a high-altitude ecosystem. (* indicates carnivorous mammals).

The three mammal species with the highest *RA* are bharal (*Pseudois nayaur*), Moupin pika (*Ochotona thibetana*), and Himalayan marmot (*Marmota himalayana*). The four bird species with the highest *RA* are the snow partridge (*Lerwa lerwa*), plain mountain finch (*Leucosticte nemoricola*)*,* Chinese monal, and alpine accentor (*Prunella collaris*) (Figure 4). The mammals are partly grouped as phyllophilic species and partly grouped as thermophilic, while the birds have the highest relative abundance in both the cold-resistant and phyllophilic groups. For thermophilic species, the relative abundance of mammals is higher than that of birds.

## 4. Discussion

### 4.1. Ethology of the Wildlife in Alpine Community

The characteristics of alpine environments include low average temperatures, strong winds, and prolonged snow covers [17]. Wildlife living at high elevations must cope with these extreme environmental conditions. Their behavior, biology, and life-history traits may reflect species’ responses to alpine environments.

Our cameras captured approximately eight wolves on the alpine scree of Weijiagou. These are the first images and videos of wolves in the WNNR. Beech martens were found to be nocturnal, and they showed collective behavior in July. This is consistent with a previous study [18] that found that beech martens were active at night and that their mating season is in July and August.

Mountain weasels (*Mustela altaica*) were widely distributed; they were active throughout the year. Our cameras captured the moments at which a mountain weasel preyed on a Moupin pika and a snow partridge, which are essential for dietary analysis. The hog badger (*Arctonyx collaris*) and Himalayan marmot hibernate in winter; hog badgers spend approximately six months per year in hibernation, while Himalayan marmots spend approximately four months in hibernation. Swinhoe’s striped squirrel (*Tamiops swinhoei*) was observed in July at an elevation of ~4200 m. Liu et al. [19] also captured swinhoe’s striped squirrel at elevations ranging from 4200 m to 4500 m. Sichuan takins were observed on alpine scree at an elevation above 4200 m in summer, which partly confirms that Sichuan takin has a vertical distribution of habitats at elevations ranging from 1500 m to 4500 m as reported in previous observations [20]. Chinese gorals typically prefer a forest habitat [21]. We observed Chinese gorals on alpine scree at an altitude of ~4200 m. This is the first report of a Chinese goral in alpine scree.

Snow partridge and Tibetan snowcock (*Tetraogallus tibetanus*) mainly stay in areas with an altitude of above 4200 m year round, which is not consistent with their seasonal vertical migration observed in previous studies [22,23]. Further research is needed to explore the reasons for this discrepancy. We found that Chinese monal, blood pheasant (*Ithaginis cruentus*) and passerine birds migrated vertically.

### 4.2. Habitat Environmental Factors Relationships

According to the results of the DCCA, bharal, wolf and snow leopard are cold-tolerant species; thus, their suitable habitats may overlap. We inferred that the bharal is the main prey of the wolf and the snow leopard, indicating that wolves may compete with snow leopards.

The abundances of the other four carnivores, i.e., beech marten, red fox (*Vulpes vulpes*), hog badger, and mountain weasel, were similar. Both red foxes and hog badgers were frequently distributed in habitats with plenty of vegetation, and they may compete for resources. According to the intersectional part of ‘cold-resistant’ and ‘thermophilic’ analysis (Figure 3a), Swinhoe’s striped squirrel and beech marten are distributed over both cooler and warmer areas, indicating that these species may have two differentiated populations occupying different habitats or they might migrate seasonally. Additional information about food sources may help clarify ecological relationships in the alpine community. With that information, we could build alpine food-web models and predicate changes in local communities.

According to the *RA* rankings of birds, cold-resistant species show the greatest abundance. For example, Chinese monal had the greatest abundance and snow partridge had the third greatest abundance followed by alpine accentor. The phyllophilic species had lower abundances than the cold-resistant species, although the plain mountain finch (*Leucosticte nemoricola*) had the second greatest abundance. The thermophilic species had the smallest abundance. Many bird species were distributed in more than one type of habitat, e.g., blue-fronted redstart (*Phoenicurus frontalis*), grandala (*Grandala coelicolor*), and plain-backed thrush (*Zoothera mollissima*).

### 4.3. The Outlook and Implication of the Camera-Trapping Approach

Each capture of the camera trapping includes one video clip and three photos taken consecutively. These video clips are not merely replications or supplementary information but are quite useful observational materials for identifying species and studying the ethological features of wildlife. However, we have not yet performed further analysis to dig into animal ethology and their function in the communities and ecosystems, which could be investigated as a next step. To improve the efficiency and accuracy of the camera-trapping approach for wildlife monitoring, advanced digital image processing techniques such as machine learning could be applied to these photos and videos, which are typical ‘big data’ with high volume and dynamical streaming accumulation.

Our monitoring and analysis of alpine biodiversity not only provide fundamental knowledge and evidence of how mammals and birds are distributed across various environmental gradients in the reserve but also present an example of collecting species information via a non-invasive observational technique in an alpine ecosystem and generalizing groups of wildlife species through multivariate statistical analyses. In addition, the quantified abundance information of wildlife can be referred to when setting out localized species conservation strategies, as well as policy and nature reserve planning. Admittedly, even at our best, in this stage of our pilot study, there is a limitation to the number and representativity of sampling sites, which restricts our analysis to the current depth. Further efforts could be made to develop models for mapping species distributions with data collection at a larger spatial range designed in a systematically gridded plan on our present basis.

## 5. Conclusions

In this study, we monitored and accessed the activities of mammals and birds in the mountainous forest in WNNR of Sichuan, China, by a camera-trapping approach. We classified the wildlife into three groups ((1) cold-resistant, (2) phyllophilic, and (3) thermophilic species) based on their responses to key environmental drivers, i.e., temperature and vegetation. We calculated and ranked the relative abundance of the observed species. The mammal species with the highest relative abundance were bharal, Moupin pika, and Himalayan marmot. The bird species with the highest relative abundance were snow partridge, plain mountain finch, Chinese monal, and alpine accentor. Our results offer fundamental knowledge on the distribution patterns of mammals and birds and their relationship with key environmental factors in the alpine ecosystems in southwestern China, which could assist further strategizing for local biodiversity conservation under environmental gradients.

## Data Availability

Not applicable.

## Supplementary Materials

The following are available online at https://www.mdpi.com/article/10.3390/ani11123377/s1, Table S1. All mammals and birds species namelist monitored by the camera-trapping in the Wolong National Nature Researve, China, Table S2. The environmental factors in the installation sites of camera-trapping in Tizi Valley, Yinchang Valley, and Weijia Vally, in the Wolong National Nature Researve, China, Figure S1. The demonstration of habitats in the infrared-triggered cameras installation sites for the snow leopards in Wolong National Nature Reserve.
