# Supplementary material for: Habitat Use and Activity Patterns of Mammals and Birds in Relation to Temperature and Vegetation Cover in the Alpine Ecosystem of Southwestern China with Camera-Trapping Monitoring
Source: Animals (Basel). 2021 Nov 25;11(12):3377. doi: 10.3390/ani11123377 (PMC8698180; doi:10.3390/ani11123377)
Supplement: Supplementary file 1 [file animals-11-03377-s001.zip › animals-1370671-supplementary.pdf]

Article

Habitat Use and Activity Patterns of Mammals and Birds in Relation to Temperature and Vegetation Cover in the Alpine Ecosystem of Southwestern China with Camera-trapping Monitoring

Supplementary Materials

Table S1. All mammals and birds species namelist monitored by the camera-trapping in the Wolong National Nature Researve, China

No.	Class	Order	Family	Species	Chinese National List of Protected Animals Category	effective detection	relative abundance
1	Mammals	Carnivora	Canidae	*Canis lupus*		1	0.14
2	Mammals	Carnivora	Canidae	*Vulpes vulpes*		30	4.25
3	Mammals	Carnivora	Mustelidae	*Martes foina*	II	18	2.55
4	Mammals	Carnivora	Mustelidae	*Mustela altaica*		69	9.78
5	Mammals	Carnivora	Mustelidae	*Arctonyx collaris*		48	6.8
6	Mammals	Carnivora	Felidae	*Panthera uncia*	I	43	6.09
7	Mammals	Rodentia	Sciuridae	*Tamiops swinhoei*		1	0.14
8	Mammals	Rodentia	Sciuridae	*Marmota himalayana*		285	40.39
9	Mammals	Rodentia	Cricetidae	*Volemys millicens*		8	1.13
10	Mammals	Lagomorpha	Ochotonidae	*Ochotona gloveri*		319	45.21
11	Mammals	Cetartiodactyla	Bovidae	*Budorcas tibetanus*	I	75	10.63
12	Mammals	Cetartiodactyla	Bovidae	*Pseudois nayaur*	II	850	120.47
13	Mammals	Cetartiodactyla	Bovidae	*Naemorhedus griseus*	II	10	1.42
14	Aves	Falconiformes	Accipitridae	*Accipiter nisus*	II	2	0.28
15	Aves	Falconiformes	Accipitridae	*Gypaetus barbatus*	I	2	0.28
16	Aves	Falconiformes	Accipitridae	*Gyps himalayensis*	II	2	0.28
17	Aves	Galliformes	Phasianidae	*Ithaginis cruentus*	II	7	0.99
18	Aves	Galliformes	Phasianidae	*Lerwa lerwa*		112	15.87
19	Aves	Galliformes	Phasianidae	*Lophophorus lhuysii*	I	106	15.02

20	Aves	Galliformes	Phasianidae	*Tetraogallus tibetanus*	II	39	5.53
21	Aves	Columbiformes	Columbidae	*Columba leuconota*		1	0.14
22	Aves	Passeriformes	Turdidae	*Phoenicurus frontalis*		28	3.97
23	Aves	Passeriformes	Turdidae	*Chaimarrornis leucocephalus*		22	3.12
24	Aves	Passeriformes	Turdidae	*Grandala coelicolor*		19	2.69
25	Aves	Passeriformes	Turdidae	*Turdus mupinensis*		13	1.84
26	Aves	Passeriformes	Turdidae	*Turdus kessleri*		1	0.14
27	Aves	Passeriformes	Turdidae	*Zoothera mollissima*		15	2.13
28	Aves	Passeriformes	Timaliidae	*Garrulax elliotii*		1	0.14
29	Aves	Passeriformes	Fringillidae	*Carduelis thibetana*		3	0.43
30	Aves	Passeriformes	Fringillidae	*Leucosticte nemoricola*		27	3.83
31	Aves	Passeriformes	Fringillidae	*Carpodacus nipalensis*		10	1.42
32	Aves	Passeriformes	Fringillidae	*Mycerobas carnipes*		2	0.28
33	Aves	Passeriformes	Corvidae	*Pyrrhocorax graculus*		3	0.43
34	Aves	Passeriformes	Corvidae	*Pyrrhocorax pyrrhocorax*		4	0.57
35	Aves	Passeriformes	Corvidae	*Corvus macrorhynchos*		1	0.14
36	Aves	Passeriformes	Prunellidae	*Prunella collaris*		62	8.79
37	Aves	Passeriformes	Prunellidae	*Prunella strophiata*		12	1.7

Table S2. The environmental factors in the installation sites of camera-trapping in Tizi Valley, Yinchang Valley, and Weijia Vally, in the Wolong National Nature Researve, China

**No.**	**site code**	**altitude (m)**	**longitde (degree)**	**Lattitude (degree)**	**aspect**	**slope (degree)**	**shrub coverage**	**herbal coverage**	**habitat type (score)**	**maximum activity tem-perature (℃)**	**minimum activity tem-perature (℃)**	**average activity tem-perature (℃)**	**distance to the closest water source (m)**
1	T1	4437	103.0406	30.84752	NW	60	0	0.2	alpine scree (4.5)	31	-11	7.1	1643.09
2	T2	4039	103.0350	30.8489	W	40	0	0.1	alpine scree (3.2)	31	-4	16.4	1561.57
3	T3	3985	103.0339	30.84865	NW	40	0.4	0.8	alpine shrub (21.9)	4	-4	-0.3	1614.75
4	T4	4211	103.0376	30.84763	NW	40	0.6	0.2	alpine shrub (24.9)	35	3	10.3	1653.77
5	T5	4481	103.0408	30.84892	S	30	0	0.2	alpine scree (4.5)	22	-3	8.9	1499.32

6	T6	4310	103.0369	30.84210	NW	50	0	0.1	alpine scree (3.2)	30	0	5.2	2299.02
7	T7	4126	103.0345	30.84316	W	50	0.4	0.5	alpine shrub (21.2)	27	1	8.8	2182.25
8	T8	4265	103.0379	30.84671	W	60	0	0.3	alpine scree (5.5)	29	-6	9.6	1763.83
9	T9	4264	103.0359	30.84146	W	40	0	0.3	alpine scree (5.5)	34	-3	10.7	2356.87
10	T10	4188	103.0368	30.84683	W	20	0	0.5	alpine scree-meadow (7.1)	29	5	13.4	1760.19
11	T11	4460	103.0403	30.84716	NW	30	0	0.2	alpine scree (4.5)	30	4	13.0	1860.45
12	T12	4432	103.0389	30.84473	SW	20	0	0.1	alpine scree (3.2)	34	-10	0.6	1978.83
13	T13	4458	103.0396	30.84574	NW	60	0.1	0.1	alpine meadow (10.5)	33	12	21.3	1721.26
14	Y1	4102	103.0225	31.08365	S	50	0	0.8	alpine scree-meadow (8.9)	27	-11	5.4	1303.29
15	Y2	4101	103.0236	31.08122	SW	40	0	0.5	alpine scree-meadow (7.1)	24	0	6.2	1356.13
16	Y3	3811	103.0140	31.08771	SW	40	0	0.8	alpine meadow (9.9)	15	-12	3.3	517.18
17	Y4	4214	103.0115	31.08652	SW	60	0	0.1	alpine scree (3.2)	33	-13	6.2	1146.91
18	Y5	3575	103.0086	31.07286	W	50	0.2	0.3	alpine shrub (15.2)	8	-1	2.8	277.35
19	Y6	3536	103.0108	31.06893	SW	60	0.2	0.2	alpine shrub (14.8)	18	-2	9.2	110.88
20	Y7	4087	103.0111	31.08158	W	40	0	0.1	alpine scree (3.2)	16	-10	6.1	994.29
21	Y8	4103	103.0122	31.08033	W	40	0	0.5	alpine scree-meadow (7.1)	32	-4	5.7	1002.46
22	Y9	3819	103.0128	31.07305	S	40	0	0.8	alpine meadow (9.9)	27	1	8.8	591.80
23	W1	4221	102.9130	30.93716	SE	30	0	0.5	alpine scree-meadow (7.1)	33	-9	8.3	1015.26
24	W2	4341	102.9109	30.93816	SE	50	0	0.2	alpine scree (4.5)	37	-2	12.4	1097.51
25	W3	4418	102.9103	30.93928	SE	50	0	0.3	alpine scree (5.5)	24	-9	9.0	1088.38
26	W4	4321	102.9116	30.93842	SE	50	0	0.3	alpine scree (5.5)	19	-6	6.4	1029.06
27	W5	4378	102.9104	30.93842	S	50	0	0.5	alpine scree-meadow (7.1)	36	2	15.8	1125.32

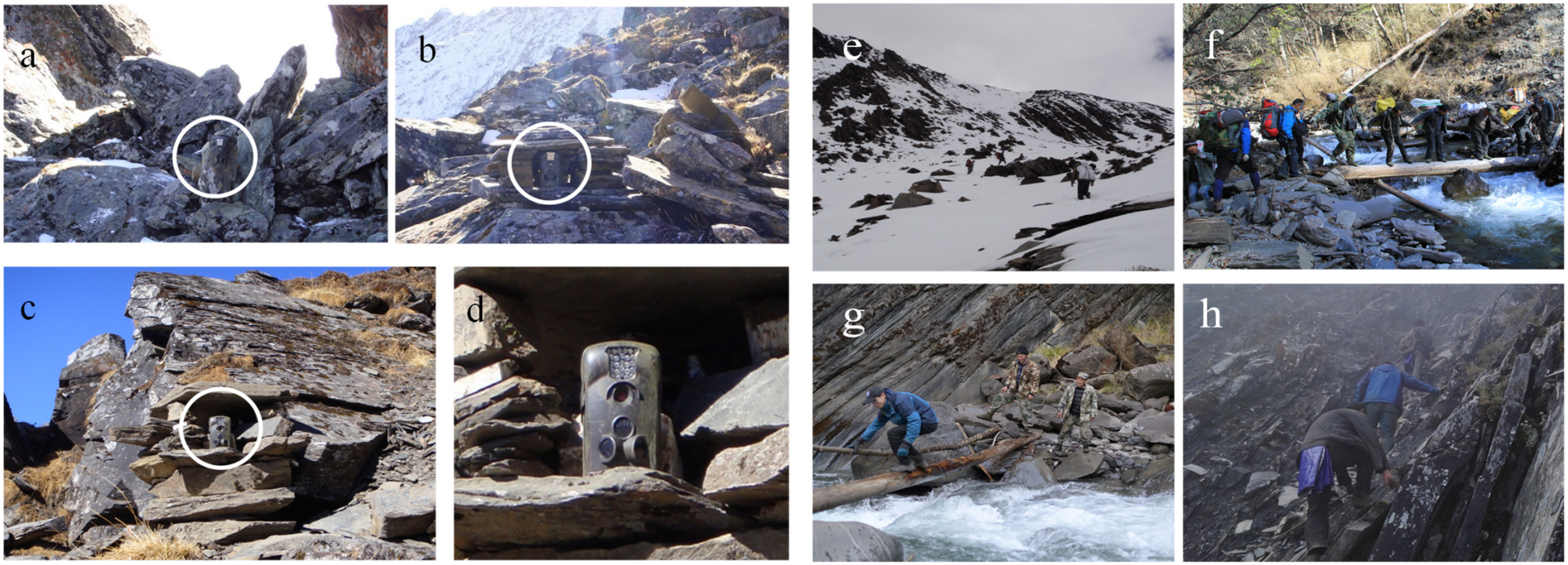

Figure S1. The demonstration of habitats in the infrared-triggered cameras installation sites for the snow leopards in Wolong National Nature Reserve. a: Tizi Valley, b: Weijia Valley, c: Yinchang Valley, d: Magnified part of figure c. e~h: The photos taken during the fieldwork of the camera installation and the habitat survey in the alpine habitats of the wildlife in the Wolong National Nature Reserve. (the man in the blue coat is Zhuo Tang, one of the leading author of this article.)
